# COVID-19-associated liver injury, role of drug therapy and management: a review

**DOI:** 10.1186/s43066-022-00230-y

**Published:** 2022-11-23

**Authors:** Chinonyerem O. Iheanacho, Okechukwu H. Enechukwu

**Affiliations:** 1 Department of Clinical Pharmacy and Public Health, Faculty of Pharmacy, University of Calabar, Calabar, Cross River State 540271 Nigeria; 2 Pharmacy Department, General Hospital Aboh, Aboh, Delta State Nigeria

**Keywords:** COVID-19, SARS-CoV-2, Liver injury, Drug induced, Pre-existing liver diseases, Clinical features, Laboratory features

## Abstract

The ongoing COVID-19 pandemic is known to affect several body organs, including the liver. This results from several factors such as direct effect of SARS-CoV-2 on the liver, side effects of drug therapy and pre-existing liver diseases. Drug-induced liver injury can result from a range of drugs used in the treatment of COVID-19 such as antiviral drugs, anti-inflammatory drugs, antibiotics, herbal medications and vaccines. Metabolism of most drugs occurs in the liver, and this leaves the liver at risk of medication-induced liver damage. Being among pathologies from the disease, COVID-19 liver injury presents with abnormally high liver-related enzymes, such as aspartate aminotransferase, alanine aminotransferase, alkaline phosphate (ALP), and gamma-glutamyl transferase. It is reversible, generally not severe and occurs more mildly in children. However, COVID-19-associated liver injury is worsened by chronic liver diseases and vice versa. There is a high risk of abnormal ALT and AST, in-hospital liver injury and prolonged SARS-CoV-2 shedding in COVID-19 patients with previously existing metabolic-associated fatty liver disease. COVID-19-associated liver injury also appears to be severe and significantly associated with life-threatening COVID-19 and mortality in persons with a history of liver transplant. Where necessary, only supportive management is usually indicated. This paper evaluates the aetiology, clinical and laboratory features, occurrence and management of COVID-19-associated liver injury. It also elaborated on the role of drug therapy in the development of COVID-19 liver injury.

## Background

The onset of coronavirus disease 2019 (COVID-19) pandemic has precipitated several human and economic losses. This is largely due to its associated high infectivity, morbidity and mortality. Among risk factors that have influenced disease progression are older age, immunodeficiency and underlying comorbidities, such as pre-existing liver disease. Although some drugs have shown positive outcomes in the management of COVID-19 [1, 2], highly effective vaccines appear to be the mainstay for curbing its spread [3]. Among the previously used drugs are hydroxychloroquine, antiviral drugs (favipiravir, lopinavir/ritonavir, umfenovir, remdesivir, etc.) and anti-inflammatory drugs (corticosteroids, immunoglobulin and immunomodulators) [2]. Following their pharmacokinetic and pharmacodynamics pathways, these drugs also possess potentials to precipitate liver injury in COVID-19 patients.

COVID-19 is associated with several pathologies, among which is liver injury which results from several factors. The severity of the resulting injury is suggestive of COVID-19 prognosis [4], including development of acute respiratory syndrome (ARDS). COVID-19-related liver injury may result from direct SARS-CoV-2 infection of the liver, systemic inflammatory response to SARS-CoV-2 infection, exacerbation of pre-existing liver disease by the infection, side effects of COVID-19 drug therapy or hypoxic changes. Other risks of liver involvement in COVID-19 is old age and disease severity [5–7]. COVID-19-related liver injury shows various degrees of abnormal liver function tests which correlate with poor disease prognosis [8]. Moderate microvesicular steatosis as well as mild inflammatory response at the lobules and portal region are predictors of COVID-19-induced liver injury [7].

Therefore, COVID-19-associated liver injury may be disease related and drug related or may result from aggravation of already existing chronic liver disease. It occurs in infants [9, 10], children [11] and adults [12] and may be associated with severe disease and elevated inflammatory markers. Hence, it is crucial to provide adequate monitoring for the occurrence of liver injury in the management of COVID-19. This article evaluates aetiology, clinical features, laboratory features and management of COVID-19-associated liver injury. It also elaborated on the role of drug therapy in the development of COVID-19 liver injury.

## Main text

### Aetiology of COVID-19 liver injury

Several mechanisms have been associated with the development of COVID-19-associated liver injury. More specifically, it results from hypoxic hepatitis which is caused by changes in the haemodynamics and oxygen delivery [8]. This creates a sharp rise in aminotransferases and causes a predisposition to other complications, such as respiratory failure, shock or cardiac failure. Other major causes of COVID-19 liver injury are hepatic ischaemia and hepatic venous congestion resulting from elevated venous pressure which predisposes the hepatocytes to more significant hypoxic injury.

Additionally, direct infection of the liver cells by SARS-CoV-2 also raises risks of liver injury [8]. The liver is one of the major organs infected by SARS-CoV-2, following its expression of the angiotensin-converting enzyme 2 (ACE) receptors. The presence of ACE 2 in the bile duct also increases the risks of bile duct dysfunction which inversely influences liver regeneration and immune response [5]. Being a receptor for SARS-CoV-2 spike (S) protein, hosts’ cell infection of SARS-CoV-2 is mediated by interaction of ACE2 and S protein [1]. Therefore, hepatic and bile duct infection occur with cleaving of S protein transmembrane serine protease 2 (TMPRSS2) to ACE2 [13].

Exacerbation of previously existing chronic liver disease by medications used in COVID-19 therapy is also implicated in the aetiology of COVID-19 liver injury. As shown in previous studies, antivirals, antibiotics and anti-inflammatory drugs (such as steroids) have been used in the management of COVID-19 [2]. Some of these drugs, including acetaminophen which is routinely used for symptomatic management of COVID-19, are metabolized in the liver. Specific drugs implicated in COVID-19 liver injury are lopinavir/ritonavir [7], remdesivir and hydroxychloroquine [14]. Pathogenic immunological reactions to COVID-19 vaccines with elevated pro-inflammatory markers have also been widely reported [15].

Cytokine storm or excessive systemic inflammatory response, usually occurring as elevated inflammatory markers, particularly interleukin-6 and ferritin, is significantly associated with development of mild, moderate and severe liver injury in COVID-19 patients [12]. Severe liver injury indicates poorer clinical course of disease, including increased rates of intensive care units, intubation and mortality [12, 16].

### COVID-19 liver injury in special populations

Chronic liver disease in children appear not to be associated with elevated risks of SARS-CoV-2 infection or severity of COVID in the population [17]. However, abnormal levels of liver enzymes in children during SARS-CoV-2 infection may relate to underlying diseases or COVID-19 drug therapy [11]. Liver injury appears to be more prevalent in infants [10] than in older children where it is uncommon and mild [11] during COVID-19 disease. Liver failure is also uncommon in children with COVID-19; however, a case report suggests a rare risk of COVID-19 associated acute liver failure secondary to type 2 autoimmune hepatitis in children [18]. This suggests adequate vigilance and monitoring of children with the disease. Meanwhile, COVID-19-induced abnormal findings from liver tests appear to be generally mild in pregnant women.

Development of liver injury significantly correlates with increased incidence of mortality and critical disease state in liver transplant recipients with COVID-19 [19]. Higher frequency of COVID-19-related symptoms is suggested in liver transplant recipients [20]. High mortality is also associated with liver and kidney transplant HIV patients with COVID-19 [21]. Therefore, close monitoring of these patients is crucial and of high priority. Younger age, metabolic syndrome, use of vasopressor and antibiotics are important determinants of liver injury in persons with liver transplantation. However, a previous study has suggested that liver function indicators remain within normal after COVID-19 vaccination in liver transplant recipients, and incidences of compromised graft function, graft rejection, neurological conditions or severe allergic reaction are not likely to occur [22]. In their studies, Tu et al. and Burra & Russo observed that immune response to the inactivated COVID-19 vaccine and mRNA SARS-CoV2 vaccine was generally low among liver transplant recipients [23, 24], and shorter time post liver transplantation is a predictor of this [23]. However, COVID-19 vaccination is generally safe in liver transplant recipients [22].

### COVID-19 in patients with pre-existing liver disease (e.g. chronic liver disease and hepatitis)

Globally, deaths due to liver diseases are estimated at 2 million per year with complications due to cirrhosis accounting for 1 million deaths [25]. Also, drug-induced liver injury appears to be on the increase, accounting for most cases of acute hepatitis [25]. A deterioration of liver function characterized by an ongoing progressive inflammation of the liver parenchyma as well as destruction and regeneration of the parenchyma occurring over 6 months or more which subsequently results in fibrosis or cirrhosis is referred to as chronic liver disease [26]. Most prevalent etiologies of chronic liver disease seen globally include non-alcoholic fatty liver disease (NAFLD) now referred to as metabolic-associated fatty liver disease (MAFLD), alcohol liver disease, chronic viral hepatitis, genetic causes (e.g. Wilson’s disease), autoimmune causes (e.g. autoimmune hepatitis), and other causes (e.g. drugs and idiopathic causes) [26].

Though the prevalence of pre-exiting liver disease in COVID-19 has been reportedly low, ranging from 3 to 8% [27], a systematic review by [28] reported a 46.9% prevalence of one or more abnormal liver function test (LFT), in COVID-19 patients at admission. Various studies have been carried out to evaluate the role of pre-existing liver disease on COVID-19 outcome and the effect of COVID-19 on previously existing liver diseases. As seen in a multinational retrospective cohort study involving over 220 patients across 13 countries in Asia, previously existing liver disease is worsened by SARS-COV-2 infection [29]. Meanwhile, the severity of COVID-19 has also been found to be increased by pre-existing liver disease [27]. Exacerbation of pre-existing liver disease may also occur from other factors such as drug-induced hepatotoxicity of commonly used medications for COVID-19 management.

### MAFLD/NAFLD

As noted in previous studies, risk of severe COVID-19 is higher in persons with pre-existing MAFLD [27, 30–32]. Although co-existing metabolic risk factors in MAFLD/NAFLD are considered risk factors for severe COVID-19 [33], an independent association exists between NAFLD and severe COVID-19 (characterized by ICU admissions) [34]. From findings in a more recent study, NAFLD basically appears to be a predictor of COVID-19-associated liver injury [35]. Consequently, there is a high risk of abnormal ALT and AST, in-hospital liver-related injury, and extended SARS-COV-2 shedding in persons with pre-existing MAFLD/NAFLD and COVID-19 [27].

### Alcohol liver disease

A range of comorbidities (e.g. chronic kidney disease, diabetes) which are independent risk factors for COVID-19 complications exist in chronic alcohol consumers who have developed alcohol-associated diseases [36]. These alcohol-associated diseases are independent risk factors for mortality in COVID-19 as seen in a previous multinational cohort study [37].

### Chronic viral hepatitis

It is hypothesized that reactivation and massive replication of hepatitis B virus (HBV) will occur in persons with a history of HBV liver disease coinfected with SARS-COV-2, which could then lead to prolonged viral shedding [27]. However, there is no significant association between chronic HBV/SARS-COV-2 coinfection and severe outcomes when compared to outcomes in COVID-19 patients with no HBV infection [38]. Therefore, chronic viral hepatitis does not necessarily elevate risks of poor prognosis of COVID-19.

### Liver cirrhosis

SARS-COV-2 is a causal factor for significant liver injury leading to worse clinical outcomes in patients with decompensated cirrhosis [39]. It also induces decompensation in persons with liver cirrhosis who are not yet decompensated [25]. There is a higher mortality rate in persons with liver cirrhosis and COVID-19 patients when compared to COVID-19 patients without liver cirrhosis, though the mortality rate is similar to that seen in patients with cirrhosis only [39].

### Role of COVID-19 drug therapy and vaccines in development of liver disease/injury

Drug-induced liver injury (DILI) can result from a range of drugs used in COVID-19 management, e.g. antiviral agents, nonsteroidal anti-inflammatory drugs, antibiotics [40] and herbal medications, e.g. Chinese herbs [41]. Metabolism of most drugs occurs in the liver, and this leaves the liver at risk of medication-induced liver damage [42]. DILI is defined as damage to the liver which occurs due to a couple of etiologies such as direct effect of a drug or its metabolite on the liver and hypersensitivity to the drug [43]. DILI can be classified in terms of duration of injury (acute or chronic DILI) as well as based on the histological location of the injury/damage (cholestatic, hepatocellular and or mixed pattern DILI) [42]. Due to the practice of polypharmacy in COVID-19 management, a focus on the role of these drugs (most of which are hepatotoxic) in the development of liver injury is essential.

A couple of factors that increase the probability of DILI include age (aged patients are more at risk), gender (men are more at risk), alcohol consumption (chronic consumption of alcohol increases the risk) and pre-existing liver disease [43]. From a meta-analysis involving 104 articles, a pooled prevalence of DILI in patients with COVID-19 was 25.4% [44]. A previous study also suggests that DILI might be responsible for the reported cases of COVID-19 liver injury seen in severe COVID-19 patients, due to the fact that such patients during hospitalization are exposed to a long course treatment involving the use of drugs that can indeed lead to DILI, e.g. antivirals, antibiotics, and steroids [40]. Mild lobule/portal region inflammation and moderate microvesicular steatosis appear to be correlated with COVID-19 drug-induced liver injury.

Elevated liver enzymes have been observed following the use of immunosuppresants (e.g. tocilizumab, dexamethasone) in COVID-19 management [40]. In patients with COVID-19 treated with tocilizumab, the drug was reported to cause an increase in serum transaminases by 40-fold which eventually resolved within 10 days [45]. This could be associated with the fact that immunosuppresants can reactivate occult hepatitis B virus (in those infected) which could result in liver injury/damage [44].

The use of the antiviral drugs: lopinavir and ritonavir, in the treatment of severe COVID-19, was reportedly associated with adverse liver effects in a randomized controlled trial [46]. In a retrospective observational study involving 148 patients, lopinavir/ritonavir was identified to independently possess risks for severe liver injury in COVID-19 patients [47]. Azithromycin, an antibiotic used in the management of COVID-19, is associated with cholestatic hepatitis, occurring within 3 weeks of initiation of treatment [48]. Based on the number of potential hepatotoxic drugs used for COVID-19 management, clinicians are expected to monitor liver function during the course of treatment, limit concurrent use of such drugs and remain pharmacovigilant.

Hepatotoxicity following the use of vaccines for the prevention of COVID-19, though rare, occurs [49]. A case of DILI in a patient who had received the COVID-19 vaccine with no other identifiable causal factor was reported in a previous study [49]. It was associated with mild increase in the level of transaminases with a marked increase in ALP and bilirubin. The DILI observed in the patient was cholestatic based on histological location classification. This therefore, stresses the need for monitoring of COVID-19 vaccine-associated adverse events as well as adverse events following immunization (AEFI) which may not necessarily have a causal association with the administered COVID-19 vaccine.

### Laboratory features of COVID-19-related liver injury

Liver function laboratory tests are made up of all indices of liver injury biochemical indicators. These include higher values of alanine aminotransferase (ALT) and aspartate aminotransferase (AST) and bile duct injury or cholestasis such as alkaline phosphate (ALP) and gamma-glutamyl transferase (GGT) [11, 50]. Bile duct damage is noted by its surrogate markers: gamma-glutamyl transferase and alkaline phosphate [51]. Higher levels of AST, ALP and ALT appear to be more prevalent at admission [4, 52]. Others are markers of liver clearance/biliary excretion capacity (total bilirubin) and indicators of synthetic capacity (which includes albumin and prothrombin time). There is no significant difference in laboratory features of males and females with COVID-19-associated liver injury [53]. AST and ALP represent better indicators of COVID-19 liver injury than ALT and total bilirubin [53].

COVID-19 liver injury mostly presents as mildly abnormal plasma liver function tests. Among the most commonly observed are elevated AST and ALT [52]. In a previous study of acute liver failure in a paediatric, it was observed to be associated with mild haemolytic aneamia, elevated lactate dehydrogenase, elevated antiliver-kidney-microsomal antibody (anti-LKM) and undetectable haptoglobin [18]. Lactate dehydrogenase occurs significantly higher in severely ill patients [54]. Other observed parameters were conjugated bilirubin, hyperammonemia and worsening coagulopathy.

Also consistent with laboratory findings in COVID-19-related liver injury is hypoalbuminaemia. Other observed pro-inflammatory cytokines are inducible protein (IP)-1 and granulocyte colony-stimulating factor (GM-CSF). Others include monocyte chemoattractant protein (MCP), IFN-γ and macrophage inflammatory protein (MIP)-1α [29]. Abnormal plasma levels of inflammatory markers are also notable in critical cases of COVID-19 [33]. Among these markers are interleukin (IL)-2, IL-7, IL-10 and tumour necrosis factor (TNF) which appear to be higher in critically ill patients than in others [33]. These may precipitated the observed liver injury.

A previous study on post-mortem liver tissue biopsy showed several pathophysiological characteristics such as microvascular steatosis and liver lobular and portal activities [55]. Among other observed abnormalities were hepatomegaly, pigmented (red) hepatocytes degeneration in addition to neutrophil infiltration and tubular degeneration. Notable entry of monocytes and lymphocytes in the portal area, as well as clogging of hepatic sinus micro-thrombosis, was also observed. Meanwhile, no evidence of liver failure or injuries of the bile duct was observed post-mortem [55].

### Clinical features of COVID-19-associated liver injury

Hepatic damage in COVID-19 patients during disease progression or treatment, regardless of the pre-existing liver injury status of a patient, is referred to as COVID-19-related liver injury [56]. Prevalence of COVID-19-associated liver injury has been recorded among severe cases of COVID-19 as against non-severe cases [57], with an estimated incidence in fatal cases ranging between 58 and 78% [58]. On the other hand, reversible liver damage has been reported with a characteristic short-term mild elevation of liver enzymes in mild COVID-19 [59]. According to Sivandzadeh et al. [60], the risk of liver injury appears to be higher in men than in women and cumulatively lower in children less than 18 years of age.

Most prevalent extrapulmonary clinical feature of COVID-19 is derangement of liver enzymes [61]. Also, findings from some meta-analyses noted that liver injury in patients with COVID-19 is essentially hepatocellular rather than cholangiocellular [60]. This is regardless of the fact that angiotensin-converting enzyme 2 (ACE2) (which is the SARS-CoV-2 receptor) is expressed more in the cholangiocytes (59.7%) compared to hepatocytes (2.6%) [62], which suggests that COVID-19-associated liver injury may be linked to cholangiocyte dysfunction [63]. More interestingly, findings from numerous studies showed that regardless of observed elevated total bilirubin levels in COVID-19 patients, presentation of jaundice is rare [60].

Elevated liver enzymes seen in COVID-19 patients from a meta-analysis were characterized by a prevalence of AST (23.2%), ALT (21.2%), GGT (15.0%), bilirubin (9.7%), and ALP (4.0%), respectively [64]. Besides the presentation of elevated liver enzymes which also serve as indicators for liver injury, there has been reported cases of darkened face and pigmentation majorly related to liver injury occurring in persons recovering from severe COVID-19 [41]. This has been linked to an increased circulating melanin and or elevated iron in the blood due to the liver injury [41].

### Management of COVID-19-associated liver injury

COVID-19-associated liver injury may be transient and often self-limiting; however, adequate attention to persons with history of existing liver disease and transplant recipients is essential. This is critical to preventing further deterioration of the liver function. ALP peak value is predictive of worse prognosis; however, liver function tests return to around baseline in COVID-19 patients who do not have pre-existing liver disease [65]. Effective and routine monitoring of liver function by appropriate liver function tests allows early detection and prompt implementation of necessary actions.

Proper evaluation of individual response to COVID-19 drug therapy is also critical to reducing the possibility of drug-induced liver injury and preventing further drug-induced hepatotoxicity. Identification and prompt withdrawal of any hepatotoxic drug, and initiation of more suitable alternative, prevent further damage while promoting the healing process.

Supportive therapy is a major step in the effective management of COVID-19 liver injury. It includes use of necessary and appropriate therapeutic agents and substitution of offending drugs. COVID-19 liver injury is not associated with significant liver function impairment or failure, hence does not require specific drug therapy. Supportive therapy depends on the severity and may include treatment with immunosuppressants and anti-inflammatory drugs such as methylprednisolone. Other potentially relevant drugs include rifaximin and lactulose [18].

## Conclusions

COVID-19 liver injury results from several factors which may be drug therapy related or disease related. It usually presents with abnormal elevation of liver enzymes, skin pigmentations and darkened face. Its presentation is generally not severe; however, close monitoring is required for patients with existing chronic liver diseases and liver transplant recipients.

## Data Availability

Not applicable.

## Abbreviations

COVID-19: Coronavirus disease 2019
SARS-CoV-2: Severe acute respiratory syndrome coronavirus-2
NAFLD: Non-alcohol fatty liver disease
ALT: Alanine aminotransferase
ALP: Alkaline phosphate
AST: Aspartate aminotransferase
GGT: Gamma-glutamyl transferase
TNF: Tumor necrosis factor
DILI: Drug-induced liver injury
MAFLD: Metabolic-associated fatty liver disease
ICU: Intensive care unit
